# The pulmonary pathology of COVID-19

**DOI:** 10.1007/s00428-021-03053-1

**Published:** 2021-02-19

**Authors:** Hans Bösmüller, Matthias Matter, Falko Fend, Alexandar Tzankov

**Affiliations:** 1 Institute of Pathology and Neuropathology, University Hospital Tübingen and Eberhard Karls University Tübingen, Liebermeisterstraße 8, 72076 Tübingen, Germany; 2 Pathology, Institute of Medical Genetics and Pathology, University Hospital Basel, University of Basel, Basel, Switzerland

**Keywords:** SARS-CoV-2, COVID-19, Diffuse alveolar damage, Pulmonary disease, Autopsy

## Abstract

The lung is the main affected organ in severe coronavirus disease 2019 (COVID-19) caused by the novel coronavirus SARS-CoV-2, and lung damage is the leading cause of death in the vast majority of patients. Mainly based on results obtained by autopsies, the seminal features of fatal COVID-19 have been described by many groups worldwide. Early changes encompass edema, epithelial damage, and capillaritis/endothelialitis, frequently combined with microthrombosis. Subsequently, patients with manifest respiratory insufficiency exhibit exudative diffuse alveolar damage (DAD) with hyaline membrane formation and pneumocyte type 2 hyperplasia, variably complicated by superinfection, which may progress to organizing/fibrotic stage DAD. These features, however, are not specific for COVID-19 and can be found in other disorders including viral infections. Clinically, the early disease stage of severe COVID-19 is characterized by high viral load, lymphopenia, massive secretion of pro-inflammatory cytokines and hypercoagulability, documented by elevated D-dimers and an increased frequency of thrombotic and thromboembolic events, whereas virus loads and cytokine levels tend to decrease in late disease stages, when tissue repair including angiogenesis prevails. The present review describes the spectrum of lung pathology based on the current literature and the authors’ personal experience derived from clinical autopsies, and tries to summarize our current understanding and open questions of the pathophysiology of severe pulmonary COVID-19.

Since the outbreak of severe respiratory infections caused by the novel coronavirus termed SARS-CoV-2 in Wuhan, China, in the end of 2019 [1], the pandemic has resulted in more than 110 million confirmed infections and more than 2.4 million deaths worldwide (February 2021), with true numbers likely much higher.

Although SARS-CoV-2 infection is considered a systemic disease and may affect virtually every part of the body, the central finding and the primary cause of death in most cases of severe COVID-19 is lung damage. Results obtained from autopsies have been crucial for the identification of pathomechanisms of lethal COVID-19 and have provided important information for treatment approaches. Despite the recent emergence of COVID-19, significant advances have already been made in understanding of this viral infection and its sequelae, although it has to be acknowledged that much has still to be learned about this novel disease. This review briefly summarizes the current knowledge of pulmonary COVID-19 pathology and pathophysiology and draws on the experience of the authors obtained from autopsies of fatal COVID-19.

## The pulmonary pathology of fatal COVID-19

In most individuals, COVID-19 is characterized by flu-like symptoms caused by the viral infection itself. A subset of patients develops acute lung injury (ALI), also called pneumonia, frequently accompanied by coagulopathy. The majority of patients with severe COVID-19 are elderly males (M:F ratio 2:1–3:1), with a mean age of 73–81.5 years in autopsy cohorts, and a range from 31 to 96 years [2, 3]. The majority of individuals show one or several comorbidities, including hypertension, atherosclerosis, ischemic cardiomyopathy and/or coronary heart disease, chronic obstructive pulmonary disease, diabetes, ATTR amyloidosis, and obesity [4–6]. Pulmonary damage is the dominant feature in most cases of severe COVID-19 and the primary cause of fatal outcome. Mainly based on autopsy series, including so-called minimally invasive autopsy using post-mortem transthoracic or transbronchial necropsy [7–9], the seminal pathological features, and the time course and evolution of pulmonary alterations, have been described, and underlying pathomechanisms are being investigated [10–16].

**Fig. 1:**
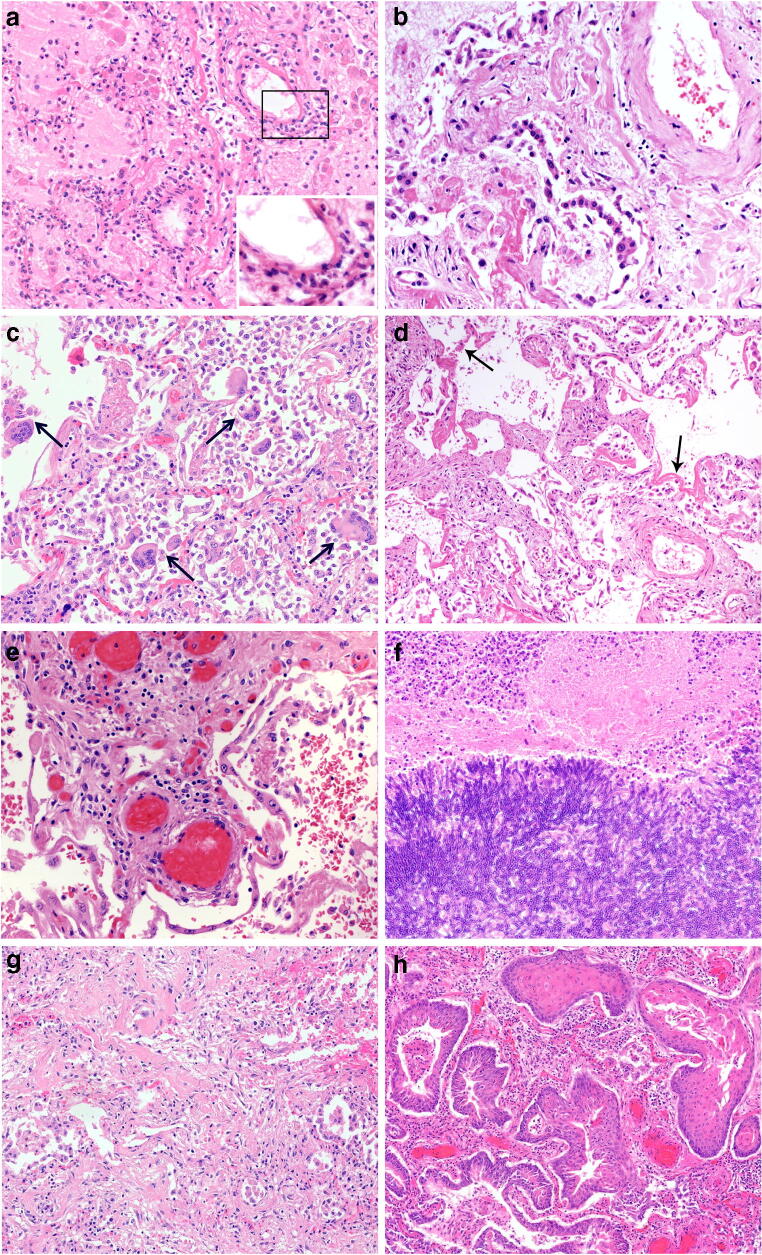
Pulmonary pathology of fatal COVID-19. **a** Incipient pulmonary damage in early-stage disease with intraalveolar edema, endothelial necrosis, microthrombosis, and endothelialitis. Insert with progressive inflammatory vascular injury with nuclear debris and granulocytes. (HE ×100) **b** Pneumocyte type 2 hyperplasia in exudative phase of DAD. (HE ×100) **c** Multiple intraalveolar giant cells in exudative phase DAD. (HE ×100) **d** Hyaline membranes and solid fibrin deposits in exudative phase DAD (HE ×100) **e** Thrombosis in small- to medium-sized vessels in early-stage lung disease (HE ×100) **f** Invasive aspergillosis as rare type of superinfection in late-stage DAD. (HE ×100) **g** Interstitial and intramural fibrosis and intraalveolar plugs of loose connective tissue in organizing phase of DAD. (HE ×100) **h** Massive bronchopulmonary squamous metaplasia in late stage organizing DAD. (HE ×100)

Pulmonary COVID-19 can be subdivided into 4 main morphological stages, including (1) an early stage (day 0–1) with edema, incipient epithelial damage, and capillaritis/endothelialitis, (2) the stage of exudative diffuse alveolar damage (DAD) (days 1–7), and (3) the organizing (1 to several weeks) and (4) the fibrotic stage of DAD (weeks to months) (Fig. 1). Although this indicates an orderly progression along the different stages, and the fibrotic stage is usually associated with long-standing severe disease, it needs to be emphasized that different DAD manifestations frequently coexist side by side, a reflection of the marked spatial and temporal heterogeneity of COVID-19 [10, 11, 17]. Among the determinants of pulmonary pathology in severe COVID-19 are time from onset of disease, comorbidities, the extent of intensive therapeutic measures including mechanical ventilation, and the presence or absence of superinfection. Descriptions of early-stage pulmonary COVID-19 are very limited (virtually based on a handful cases) and include incidental findings in lobectomy specimens from patients undergoing surgery for lung cancer. Focal edema, epithelial damage and pneumocyte hyperplasia, desquamation of alveolar macrophages, and capillaritis/endothelialitis were among the reported findings, although it is not clear whether these are related to SARS-CoV-2 infection [18, 19]. In the few published fatal cases early after infection, neutrophilic capillaritis, capillary microthrombosis, massive pulmonary edema, and signs of early epithelial damage but little interstitial or alveolar inflammation were observed (Fig. 1a) [12, 20]. These pulmonary microvascular changes are an important feature of COVID-19, and may contribute to hypoxemia and acute cardiac failure [21].

Gross examination of the lungs usually shows an increased weight with edema and diffuse congestion, and cut surfaces with irregularly distributed regions of consolidation, and—in a subset of cases—areas of hemorrhage or infarction, frequently with visible thrombosis in feeder vessels [11]. Of note, infarcts frequently lack the typical wedge shape, possibly due to thrombotic occlusion of multiple vessels rather than a single embolus. Pleural effusion is uncommon in COVID-19 [17].

Histologically, in the exudative phase of DAD, the alveolar spaces contain fibrin-rich edematous fluid, an increase in macrophages with occasional multinucleated giant cells of epithelial origin, but without viral inclusions, and variably prominent hyaline membranes (Fig. 1c, d) [12, 16, 22]. Intraalveolar hemorrhage is frequently observed, usually in association with thrombotic vessel occlusions and/or superinfections (Fig. 1e, f). Areas of dilated alveolar ducts and collapsed alveoli can occur side by side. Epithelium shows frequent necrosis, associated with marked type 2 pneumocyte hyperplasia, including atypical regeneration (Fig. 1b). A common feature are platelet–fibrin thrombi in capillaries and small arterial vessels, with occasional intravascular megakaryocytes [23], sometimes accompanied by significant vascular inflammation. Of note, neither the increase in intrapulmonary megakaryocytes nor microthrombotic disease is specific for COVID-19, but has been observed in DAD of other causes [23, 24]. According to a recent summary of published autopsy cases, microthrombi were observed in 57% of COVID-19, 58% of SARS, and 24% of H1N1 cases [24]. Thrombosis of large and intermediate-sized vessels, mostly arteries, associated with diffuse endothelial damage is a prominent but again not a specific feature of COVID-19 (Fig. 1e) [11–13, 24]. In addition, pulmonary embolism associated with deep venous thrombosis as a result of the pro-coagulatory state in COVID-19 has been described in up to 20% of patients and may be the direct cause of death [13, 25, 26]. Pulmonary infarction has been reported in 15–75% of patients in autopsy series [22, 24, 26].

Bronchopneumonia as an indication of bacterial or less commonly fungal superinfection has been described in 32–57% of patients in the larger autopsy series [22, 24, 26], although it is currently unclear how frequently superinfection has to be regarded as cause of death.

Data on the composition of the inflammatory cell component are in part contradictory, probably reflecting both the lack of standardized, systematic examinations and the significant heterogeneity of the host response. The exudative phase is represented by CD3-positive lymphocytes and occasional plasma cells, infiltrating the interstitial space; a large number of CD68-, CD163-, and CD206-positive macrophages are mainly localized in the alveolar lumina [27, 28]. Granulocytes are usually involved in vascular thromboinflammation and formation of neutrophilic extracellular traps (NETs; see later), but are not a dominant population in the alveoli, unless there is superinfection. Using a variety of analytical platforms, several studies have highlighted the temporal and spatial heterogeneity of the inflammatory response in COVID-19 and defined two basic patterns. The first is considered the earlier disease phase and is characterized by high viral load, high expression of interferon pathway genes, and a dominance of M1-like macrophages, where the second pattern with low viral load is more heterogeneous in terms of gene expression profile and cellular composition, reflecting differences in evolution of DAD [29, 30]. Of note, much of what is described above in terms of tissue damage, remodeling, and inflammation is not specific for COVID-19 but rather a reflection of the specific type of ARDS with different etiologies.

Late-stage DAD is characterized by organizing changes merging into interstitial myofibroblastic proliferations, septal collagen deposition, development of loose alveolar plugs of fibroblastic tissue, and mural fibrosis (Fig. 1g). At this stage, bronchopulmonary metaplasia with squamous cells is frequently observed (Fig. 1h) [11, 31]. Of note, the 3 patterns described above can occur simultaneously in different areas of the lung and do not reflect a consistent chronological evolution. This spatial heterogeneity and the frequent presence of thrombosis and pulmonary embolism somehow limit the diagnostic value of minimally invasive autopsy described above.

## Correlation with clinical and radiological features

Although the correlation of pathological findings with clinical and radiological characteristics is well beyond the scope of this review, knowledge of the seminal features is important for the pathologist. As described below in more detail, severe COVID-19 is characterized by virally induced hyperactivation of the innate immune system resulting in a cytokine storm in early phases of the disease, with striking increases of C-reactive protein, IL1-β and IL-6 [32, 33], lymphopenia, and a profound vascular dysfunction resulting in hypercoagulability and thromboinflammation, reflected by increased D-dimer levels in almost all patients and an increased incidence of both venous and arterial thrombosis and pulmonary thrombembolism [34]. The late stage of the disease is dominated by DAD and its complications, including progressive respiratory insufficiency and frequent superinfections [29, 30, 32].

The most frequent radiological manifestation of COVID-19 is ground-glass opacities (GGO) [35]. GGO is defined as increased attenuation on chest CT, which does not obscure the bronchovascular structures. Bilateral lung involvement is typical, and the right lower lobe is the most commonly affected area. Since the pathogen is inhaled with respiratory droplets, and pulmonary infection may be reinforced through active viral replication in the upper and lower respiratory tract [36], the disease is usually in bronchocentric distribution. As the disease progresses, GGO may disappear or may become more confluent and widespread and evolve into frank consolidation. Histology correlates with the imaging patterns. GGO with reticular interstitial thickening in CT is associated with mid-phase DAD, whereas the consolidation pattern is mainly associated with late-phase DAD. Our own observations suggest that GGO and consolidations correlate with multiple pathologic processes, notably DAD, capillary dilatation and congestion, and microthrombosis. Acute superposed bronchopneumonia is more frequently associated with bronchial wall thickening and consolidation and vascular enlargement sign; capillary dilatation and congestion are tightly linked to microthrombosis [37]. Discrepancies between histologic and CT findings may in part be explained by lapse between CT scan and death or superimposed bronchopneumonia [38].

## SARS-CoV-2

SARS-CoV-2, the causative agent of COVID-19, belongs to the family of coronaviruses, single-stranded positive-sense RNA viruses, with a diameter of 80–120nm [39]. Two other coronaviruses, severe acute respiratory syndrome coronavirus (SARS-CoV) and Middle East respiratory syndrome coronavirus (MERS-CoV), can also cause ALI resulting in adult respiratory distress syndrome (ARDS). Infections show many similarities in clinical presentation and pathological findings. In the few autopsies of patients, who died from SARS, the predominant pattern of ALI was DAD, including exudative and proliferative phases, inflammatory infiltrates, edema, pneumocyte hyperplasia, and fibrinous exudate. Similar to COVID-19, a prominent vascular endothelial injury and extensive ALI has been observed. Autopsy studies of patients, who died from MERS, are very limited. The ALI is characterized by exudative DAD, pneumocyte hyperplasia, and septal inflammatory infiltrate [40].

### Viral entry and the cellular tropism in the lung

**Fig. 2:**
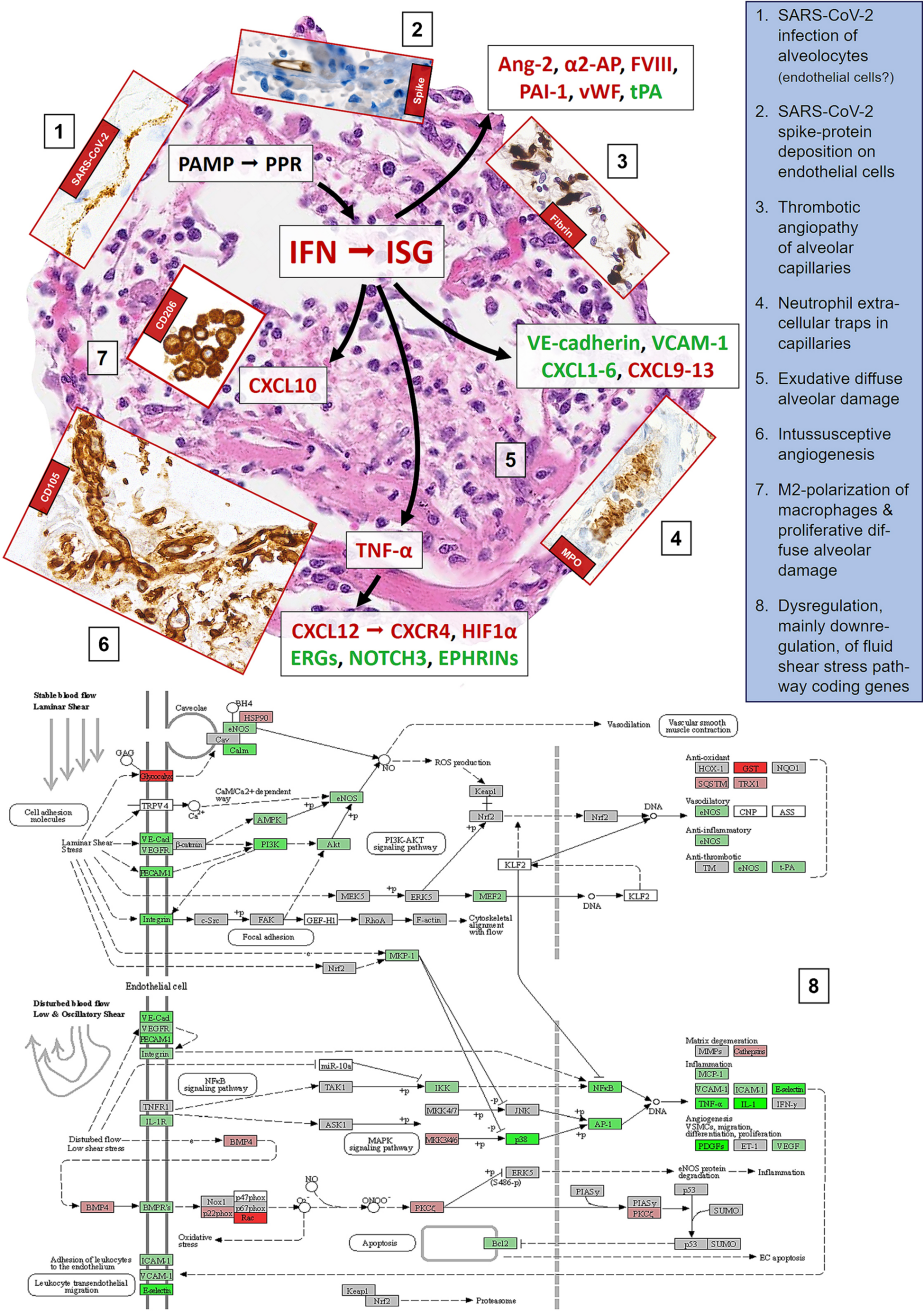
Scheme of the putative pathophysiological mechanisms of SARS-CoV-2 induced acute lung injury, mainly diffuse alveolar damage (background microphotograph (5); H&E, ×200), and microangiopathy and immunopathology in lethal (severe) COVID-19. Inserts, except for (1), are visualized by immunoperoxidase, microphotographed at ×400, and represent (1) SARS-CoV-2 in situ hybridization with the 845701 RNAscope probe - V-nCoV2019-S-sense and visualized with the RNAscope 2.5. LS detection kit (brown) from Advanced Cell Diagnostics (Hayward, CA, USA), yielding linear positivity of an alveolar septum; (2) immunohistochemical staining for SARS-CoV-2 Spike protein with the clone 007 from Sino Biological (Wayne, PA, USA), showing protein deposition on the inner side of an alveolar capillary; (3) immunohistochemical staining for fibrin with a polyclonal antibody A0080 from Dako (Glostrup, Denmark), revealing fibrin microthrombi casting the alveolar capillary network; (4) immunohistochemical staining for myeloperoxidase with a polyclonal antibody 760-2659 from Roche/Ventana (Rotkreuz, Switzerland), with neutrophilic granulocytes stuck into an alveolar capillary and displaying microscopic figures suggestive of neutrophilic extracellular traps (NET); (6) immunohistochemical staining for CD105 with the clone EPR10145-10 from Abcam (Cambridge, UK), showing a tight network of newly formed vessels in an alveolar septum; (7) immunohistochemical staining for CD206 with the clone E2L9N from Cell Signaling (Danvers, MA, USA), with significant amounts of intraalveolar M2 macrophages. (8) Kyoto Encyclopedia of Genes and Genomes (KEGG) diagram of disturbed fluid share stress pathways in COVID-19; respective gene expression profiles have been obtained on 25 lethal COVID-19 cases and compared to lungs of 5 patients suffering from arterial hypertension and 5 histopathologically unremarkable autopsy lungs that served as controls, utilizing the HTG EdgeSeq Oncology Biomarker Panel (HTG Molecular Diagnostics, Tucson, AZ, USA). The scheme should be looked at clockwise from 11 to 7. In general, upregulated genes/proteins are outlined in red, downregulated in green, indifferent ones in black

SARS-CoV-2 enters the host cell through the interaction of angiotensin-converting enzyme 2 (ACE2) with the viral spike (S) protein supported by the activity of the transmembrane protease serine subtype 2 (TMPRSS2) [41]. This allows SARS-CoV and SARS-CoV-2 to predominantly infect ciliated bronchial epithelial cells [42] and, by extension, type 2 pneumocytes (Fig. 2) as has previously been shown for SARS-CoV [43]. Consequently, virus particles are mainly detected in the epithelium of the upper respiratory tract and in the lung tissue [44]. They are mainly localized along plasmalemmal membranes and within cytoplasmic vacuoles, as described for other coronaviruses. The predominant infected cell types are type 2 and type 1 pneumocytes. In line with previous reports showing endothelial expression of ACE2 [42], viral inclusion particles have been detected in endothelial cells as well [20, 21, 28, 45]. Though this finding is still discussed controversially, as shown in Fig. 2, SARS-CoV-2 S proteins (without spike RNA) are present on endothelial cells especially with the Sino Biological antibodies clone 40150-R007 and the polyclonal one 40150-T62[46, 47], which—though being a matter of discussion [48]—may provide another potential pathogenetic mechanism of COVID-19 endothelialitis [49].

The evidence to date suggests that the viral load in the respiratory tract samples peaks around symptom onset and decreases within 1 to 3 weeks. Although the duration of detection and the viral load differs between patients, viral RNA generally becomes undetectable (from upper respiratory tract specimens) about 2 weeks after symptom onset (median 14.5 days). For lower respiratory tract samples, there is conflicting evidence regarding the timing of peak viral loads and duration of virus detection, with some evidence suggesting that it occurs later and the duration of detection is longer compared with upper respiratory tract samples (median 15.5 days) [50]. Indeed, there is some evidence that viral RNA may still present in the lungs when throat swabs become negative [51].

RNA tests can confirm the diagnosis of SARS-CoV-2 (COVID-19) cases with real-time RT-PCR or next-generation sequencing. At present, nucleic acid detection techniques are considered an effective diagnostic method in clinical cases of COVID-19 [52, 53]. More recently, other advanced diagnostics have been designed and developed for the detection of SARS-CoV-2. A reverse transcriptional loop-mediated isothermal amplification (RT-LAMP) has been established for rapid and colorimetric detection of this virus. RT-LAMP is simple and does not require sophisticated equipment or skilled personnel, but is less sensitive than RT-PCR [52, 54].

The lower respiratory tract sampling techniques such as bronchoalveolar lavage fluid and fiber bronchoscope brush biopsy are considered ideal clinical materials for critically ill patients, due to their higher positive rate on the nucleic acid test [33]. In autopsy lung tissues, the extent of lung damage does not convincingly show a correlation with the virus load, with a tendency to lower viral RNA copy numbers in late-stage disease [20, 22]. For morphological detection of SARS-CoV-2 in tissues, immunohistochemical stains for the spike glycoprotein and membrane and/or envelope proteins and viral RNA identification using the highly sensitive RNAscope technique have been developed [55, 56].

## Pathomechanisms of pulmonary COVID-19

At early stages of the pandemic, post-mortem examinations of COVID-19 patients revealed DAD with severe capillary congestion, microthrombosis, and thrombosis of small to mid-sized arteries as well as pulmonary thromboembolism suggesting vascular dysfunction [12, 57–59]. These features may explain the more specific ARDS phenotype observable in coronavirus-induced ALI with its dissociation between the relatively well-preserved lung mechanics and the severity of hypoxemia, which has been suspected to be caused by impaired regulation of pulmonary blood flow [60]. In this section, we will summarize what has been recently elucidated respecting the pathophysiology of SARS-CoV-2 interaction with the lung parenchyma and the cascade of local COVID-19 inflammatory response and endothelial and vascular damage leading to deleterious DAD, thromboinflammation, neutrophilic and macrophage dysfunction, immunopathology, and intussusceptive angiogenesis. Nevertheless, we are aware of the fact that the pathophysiology of pulmonary COVID-19 is a young and rapidly evolving field, and much of the information given below is likely to be modified with ongoing research. Furthermore, many of the pathogenetic events and mechanisms are likely not specific for COVID-19, but may be found in ALI of other causes, including other viral infections.

The virus itself is directly involved in the pathogenesis of pulmonary disease; e.g., binding of viral spike protein reduces ACE2 expression and *per se* drives ALI, leading to the excessive production of angiotensin II, which in turn increases pulmonary vascular permeability [61, 62]. Whether due to direct viral infection of endothelial cells, as discussed above, or related to immunopathology with subsequent activation of thrombotic pathways, the vasculature of the lung appears to be crucial in the pathogenesis of COVID-19, as has been recently reviewed [63], and leads to the characteristic microthrombosis of the capillary network of the alveoli (Fig. 2) and thrombosis of pulmonary arteries/arterioles.

### Hyperactivation of innate immunity

Virally induced hyperactivation of innate immunity at the level of respiratory membranes is suggested to play a major role in so-called hyper-inflammatory ARDS [64]. As in other viral infections, the innate immune response against SARS-CoV-2 is initiated by pathogens and their associated molecular patterns (PAMP) that are recognized by pattern recognition receptors (PPR), such as Toll-like receptors 3, 7, and 8 (TLR3, TLR7, TLR8) on airway epithelial cells, tissue resident macrophages, and dendritic cells [65]. These cells then initiate inflammatory pulmonary response, mainly by an upregulation of type I interferons (IFN) and interferon-stimulated genes (ISG), which is quite pronounced in early high-viral-load disease [30]; importantly yet beyond of the scope of this review, impairment of type I IFN antiviral response seems to be crucial for COVID-19 severity [66, 67]. This response can further foster epithelial and endothelial damage, as reviewed in [68], e.g., by negatively impacting on cell adhesion junctions, particularly on VE-cadherin, resulting in “loosening” of the bonds between endothelial cells [69] that leads to increased vascular permeability, a key feature of exudative ARDS [70]. Indeed, pulmonary edema and capillarostasis are the most commonly observable microscopic changes in COVID-19 [12], and *CDH5* (encoding for VE-cadherin) is among the downregulated genes in COVID-19 [21]. In addition, the ability of pneumocytes to absorb the excess fluid from the alveolar space through active sodium transport is impaired leading to edema [71]. The edema fosters hyaline membrane formation and surfactant dysfunction in exudative DAD, which is typically followed by hyperplasia of type 2 pneumocytes, initiating the proliferative phase of DAD [70]. Thus, these pneumocytes become functionally impaired to produce surfactant [72] on top of being additionally severely compromised being infected by SARS-CoV-2. This may explain the proposed link virus- especially coronavirus-induced DAD and antiphospholipid syndromes [73, 74], observable in some COVID-19 patients [75, 76] that may also directly connect ALI to coagulation.

### Endothelial and vascular damage

There is substantial evidence of profound endothelial damage in COVID-19. Coagulation factor VIII, von-Willebrand-factor (vWF), and angiopoietin 2 (Ang-2), which are stored in the Weibel-Palade bodies of endothelial cells and are released upon cell injury [77], are increased in COVID-19 [63]. Ang-2 (Ang-2) serves as an antagonist of Ang-1 and inhibits its antiinflammatory, anticoagulatory, and antiapoptotic signaling [78]; importantly, Ang-2 is directly linked to ALI and increases pulmonary vascular permeability [79], and its levels correlate with prognosis in COVID-19 [80]. COVID-19 patients have significantly elevated vWF [81], which can spontaneously bind platelets and lead to microthrombosis, and may mechanistically link the overrepresentation of blood group A individuals among severe COVID-19 [12, 82], as vWF levels are known to be higher in such individuals. Thus, it is not surprising that COVID-19 patients often initially present with increased fibrinogen levels, increased D-dimers, and suffer—as described above—from significant thrombotic complications [83]. The damaged endothelial cells lose their ability to maintain physiological functions, especially their antithrombotic activity at the luminal surface (e.g., CD39, prostaglandin 2, tissue factor pathway inhibitor), their production of tissue plasminogen activator, and NO [63]. Interestingly, there is a statistically significant link between decreased COVID-19 mortality and the distribution of improved-productivity *NOS3* haplotypes (the gene encoding for endothelial nitric oxide synthase) [84]. On top of losing its antithrombotic properties, the endothelium activated by inflammatory signals exerts an opposite battery of functions [85] by actively producing thromboxane, antiplasmin, and plasminogen activator inhibitor-1 (PAI-1). Indeed, the genes *SERPINF1* and *SERPINE1* encoding for α2-antiplasmin and PAI-1 are among the top upregulated genes in lethal COVID-19 [21]. Thus and irrespective of potential direct virus-cytopathic effects on endothelial cells, extensive thrombosis of pulmonary vessels is a predictable and consistently detectable finding. Such thrombosis would be expected to disrupt the physiological gas exchange, contributing to COVID-19-associated ALI with its characteristic hypo- to normocapnic hypoxia/“happy hypoxia” [86]. CO_2_ diffuses about 20 times more easily through tissues than O_2_ and is transported in the blood mainly (80%) as bicarbonate dissolved in plasma, the flow of which is less affected in COVID-19.

A further aspect first noticed by Magro et al. [87] features complement deposition together with neutrophil infiltration in the interalveolar septal space. Indeed, it was highly likely that complement-mediated microangiopathy might be involved in COVID-19, as it has been involved in SARS-CoV-associated ALI and in ARDS in general [88, 89]. The complement system further contributes to increased permeability of pulmonary capillaries, with subsequent fluid leakage in the alveolar space, recruitment of immune cells, and stimulation of pro-inflammatory cytokine production. Interestingly, while *SERPING1* encoding for the C1 esterase inhibitor is generally upregulated in COVID-19, *C1QA*, *C1QB*, *C2*, and *C3* encoding for the respective complement compounds are upregulated, in late low-viral-load instances in particular [21, 30]. The generated C5a is a potent chemotactic agent that leads to neutrophilic recruitment and increased vascular permeability in ALI [90]. Thus, the pathogenesis of lethal COVID-19 features a self-perpetuating process of thromboinflammation that links endothelial injury of the alveolar capillary network and other lung vessels to DAD, thrombotic microangiopathy, thrombosis of small- to mid-sized arteries, and staffing of inflammatory cells [91]. The latter, particularly neutrophils, play a key role in further (collateral) damage by releasing several toxic mediators [70] such as reactive oxygen species, and generation of NET [92]. These NET, which consist of DNA and citrullinated histones, mount antimicrobial effects and are found to cause severe tissue injury, coagulopathy, and barrier dysfunction in ARDS in general [93], and in SARS-CoV-2-induced ALI in particular [94]. Importantly, one of the NET markers, MPO-DNA correlates with elevated absolute neutrophilic count (one of the best predictors of disease severity), decreased PaO_2_/FiO_2_ ratio, a need for mechanical ventilation, and death in COVID-19 [95]. Dysregulated NETosis has been shown to also cause immunothrombosis in response to other respiratory viruses [93] by prothrombotic reprogramming of endothelial cells [96] and trapping the tissue factor pathway inhibitor.

Taken together, these findings point to the central role of vascular damage in the pathogenesis of COVID-19-related ALI as well as COVID-19-related organ damage in general that is meanwhile confirmed by multiple levels of evidence [97].

### The role of macrophages in lung injury

Resident macrophages of the lungs and those recruited from the circulation play an essential role in ARDS initiation, development, and resolution [70]. They are the primary sensor cells of PAMP through their PPR, which lead to increased intracellular BTK phosphorylation and NF-κB signaling in the context of COVID-19 [98], and are one of the most important sources of e.g. CXCL8, which is a potent chemoattractant for neutrophils (*CXCL8* being overexpressed in high-viral-load COVID-19 [21]). Macrophages are well known to terminate and resolve inflammatory ALI and coordinate structural and functional parenchymal repair processes that are essential for return to homeostasis [99]. In proliferative DAD, the macrophages shift from an M1 to an M2 phenotype to secrete antiinflammatory cytokines and to clear debris [100]. In line with the known role of these cells in ALI, bronchoalveolar lavages of patients with severe COVID-19 contained higher proportions of macrophages, yet surprisingly most of them were monocyte-derived macrophages instead of alveolar macrophages, which indicates a possible macrophage activation [101, 102]. These macrophages express chemokines (CCL2, CCL3, and CXCL10) and inflammatory transcription factors (STAT1, STAT2, ISG), which we similarly observed in high-virus-load (early) COVID-19 autopsy lungs. Importantly, the genes encoding for CXCL10 and its receptor CXCR3, which are highly chemotactic for (particularly M1) macrophages, are among the most consistently upregulated in high-virus-load COVID-19 [21, 30], while in low-virus-load, late-stage COVID-19, there is a distinct upregulation of *CD163*, *SPP1*, *TGFBI*, and *F13A* that are all considered markers of M2 macrophages. In line, a subset of alternative M2 macrophages expressing especially profibrotic genes (*TREM2*, *TGFB1*, and *SPP1*) was also identified in patients with severe COVID-19 [101]. All these findings suggest a significant pathogenic role of macrophages/macrophage activation in critical COVID-19 cases that may extend beyondinflammation [103], paving the way towards pulmonary fibrosis (see below) and macrophage activation syndrome/hemophagocytosis [104]. Indeed, compared to control lungs from various inflammatory conditions, COVID-19 autopsy lungs contain high amounts of CD163^+^ and CD206^+^ (all likely M2) macrophages (Fig. 2), and the draining lymph nodes contain high amounts of histiocytes with hemophagocytic activity and some COVID-19 patients suffer from hemophagocytic lymphohistiocytosis [105, 106].

### Cytokine dysregulation and vascular remodeling

Most data implicate cytokine dysregulation as a central factor in COVID-19 immuno(patho)logy, extending beyond the scope of this review [107]. Aberrant IFN-γ release has been previously shown to lead to epithelial and endothelial cell apoptosis and vascular leakage, suboptimal T cell response, accumulation of alternatively activated macrophages, altered tissue homeostasis, and ARDS [108]. Similarly, dysregulated TNF-α release results in apoptosis-promoting effects on highly activated effector T cells [109], and also increases the adhesion of lymphocytes in ALI by activating the SDF-1(CXCL12)/CXCR4 pathway [110], possibly also explaining decreased T cell counts (lymphopenia) in severe COVID-19. Importantly and again linking cytokines to vascular damage, CXCR4/CXCL12 signaling has been identified as potential molecular regulator of intussusceptive angiogenesis [111]. This is a unique, rapid, VEGF-independent mechanism of angiogenesis by splitting vessels into two separate lumina, incorporating circulating angiogenic cells. It acts as a physiological reaction to maintain blood flow in prolonged inflammation and is uniformly observable in early and late COVID-19 [21] (Fig. 2). Intussusceptive angiogenesis may represent a common pathophysiological denominator linking the still inestimable long-term COVID-19 complications to fibrotic interstitial lung disease [112], and—given the number of genes encoding for proteins involved in scarring that are upregulated in COVID-19 [21]—this might truly be highly relevant. Indeed, *CXCR4* is among the upregulated genes of both high- and low-viral-load COVID-19 [30], while *EPHRIN* genes, *ERG*, *EGR3*, and *NOTCH3* encoding for important regulators of sprouting angiogenesis, are downregulated in COVID-19 (own unpublished data and [21]). Blood flow, whether stable laminar or disturbed low/oscillatory (Fig. 2, part 8), is highly dependent on the morphology of the blood vessels and defines vascular shear stress [113]. While high fluid shear stress in laminar flow is angioprotective promoting endothelial cell survival, vasodilation, and anticoagulation, low shear stress results in vasoconstriction, platelet aggregation, coagulation, and pathological reshaping of microvascular architecture [112]. Indeed, analysis of known genes encoding for proteins involved in fluid shear stress suggests profound dysregulation in COVID-19 that may warrant future investigation.

## Conclusion

The COVID-19 pandemic has highlighted the critical importance of histopathologic examination of tissues, especially in the setting of clinical autopsies, for elucidating pathomechanisms and underlying causes of death in emerging diseases. Given the massive extent of the pandemic, significant efforts have been made to maximize the information gained from post-mortem examinations in fatal COVID-19. International collaborative networks and autopsy registers aim at speeding up the rate of generation and dissemination of knowledge about this novel disease [114]. A better understanding of pathophysiology, to which tissue-based examination can substantially contribute as emphasized in this review, is of prime importance for improving therapies and prognosis of patients with severe COVID-19.
